# The Effects of Resuscitative Fluid Therapy on the Endothelial Surface Layer

**DOI:** 10.3389/fvets.2021.661660

**Published:** 2021-05-07

**Authors:** Lisa Smart, Dez Hughes

**Affiliations:** ^1^School of Veterinary Medicine, College of Science, Health, Engineering and Education, Murdoch University, Murdoch, WA, Australia; ^2^Department of Veterinary Clinical Sciences, Faculty of Veterinary and Agricultural Sciences, Melbourne Veterinary School, Werribee, VIC, Australia

**Keywords:** endothelium, glycocalyx, shock, fluid therapy, crystalloid, colloid, endothelial surface layer

## Abstract

The goal of resuscitative fluid therapy is to rapidly expand circulating blood volume in order to restore tissue perfusion. Although this therapy often serves to improve macrohemodynamic parameters, it can be associated with adverse effects on the microcirculation and endothelium. The endothelial surface layer (ESL) provides a protective barrier over the endothelium and is important for regulating transvascular fluid movement, vasomotor tone, coagulation, and inflammation. Shedding or thinning of the ESL can promote interstitial edema and inflammation and may cause microcirculatory dysfunction. The pathophysiologic perturbations of critical illness and rapid, large-volume fluid therapy both cause shedding or thinning of the ESL. Research suggests that restricting the volume of crystalloid, or “clear” fluid, may preserve some ESL integrity and improve outcome based on animal experimental models and preliminary clinical trials in people. This narrative review critically evaluates the evidence for the detrimental effects of resuscitative fluid therapy on the ESL and provides suggestions for future research directions in this field.

## Introduction

The importance of microcirculatory function and health of the endothelium has become a large area of interest for criticalists in the last two decades. Over this time, fluid resuscitation strategies have experienced a shift in perspective. Aggressive “clear” fluid resuscitation was once considered vital for stabilization of macrohemodynamic variables. However, in certain patient cohorts this approach has now been shown to either not improve outcome or worsen outcome (1–4). Though fast administration of fluid often serves to normalize the commonly measured clinical parameters during shock, microcirculation may not necessarily benefit from this therapy. There is growing evidence that fluid resuscitation may actually harm the endothelium by modifying, or shedding, the endothelial surface layer (ESL).

The ESL includes a structural scaffold, the endothelial glycocalyx (EG), and associated molecules suspended within a plasma layer. We use the term *EG* when referring to specific components of the structure, or biomarkers of these components, whereas ESL is used when referring to the layer as a whole. In regards to shedding of the ESL, the term *ESL* will be used when the evidence supports loss of the whole layer and the term *EG* used when the evidence only supports that isolated components have been shed. Use of the term *glycocalyx* refers to any type of cell surface glycocalyx and is not restricted to the endothelium, such as during discussion of glycocalyx shedding biomarkers.

Resuscitative fluid therapy includes a range of fluid choices, including isotonic crystalloid fluid, synthetic colloid fluid, and hyperosmolar crystalloid fluid, with the former being the most common type of fluid used (5). In general, due to the pharmacodynamic properties of these fluids in either healthy subjects or shock models (6–9), the volume of fluid is “large” for isotonic crystalloid (at least a quarter to half of estimated blood volume), “moderate” for synthetic colloid (an eighth to quarter of estimated blood volume), and “small” for hyperosmolar crystalloids. All three types of fluid resuscitation will be covered in this review with a particular focus on isotonic “large-volume” crystalloid fluid therapy due to the evidence for its effects on the ESL. A summary of the evidence reviewed in this article is provided in Box 1.

Box 1 **Summary of the evidence for effects of “clear” fluid therapy on endothelial surface layer (ESL) shedding or modification.**

**Proposed mechanisms of ESL shedding**

Dilution of plasma proteins

Release of natriuretic peptides

Inflammatory cytokine release (certain fluid types)

**Possible downstream effects**

Exacerbation of inflammation

Microcirculatory dysfunction

Increased vascular permeability

Increased interstitial edema

Prothrombotic conditions

Proposed strategies that may mitigate ESL shedding^*^

Reduction in dose of clear fluids

Slowing down administration of resuscitative fluids

“Earlier” vasopressor therapy for vasodilatory shock

Adjunctive protein administration (such as plasma)

^*^These proposed strategies are not based on evidence from clinical veterinary research and require further investigation. These guidelines are opinion of the authors only, after considering the breadth of evidence available. It is currently unknown if “glycoprotective” strategies benefit patients. Therapy should always be tailored to individual patient needs, with prioritization of reestablishing adequate perfusion and a thorough cost to benefit analysis.

## Structure and Function of the Endothelial Surface Layer

### Basic Structure and Function

**Figure 1:**
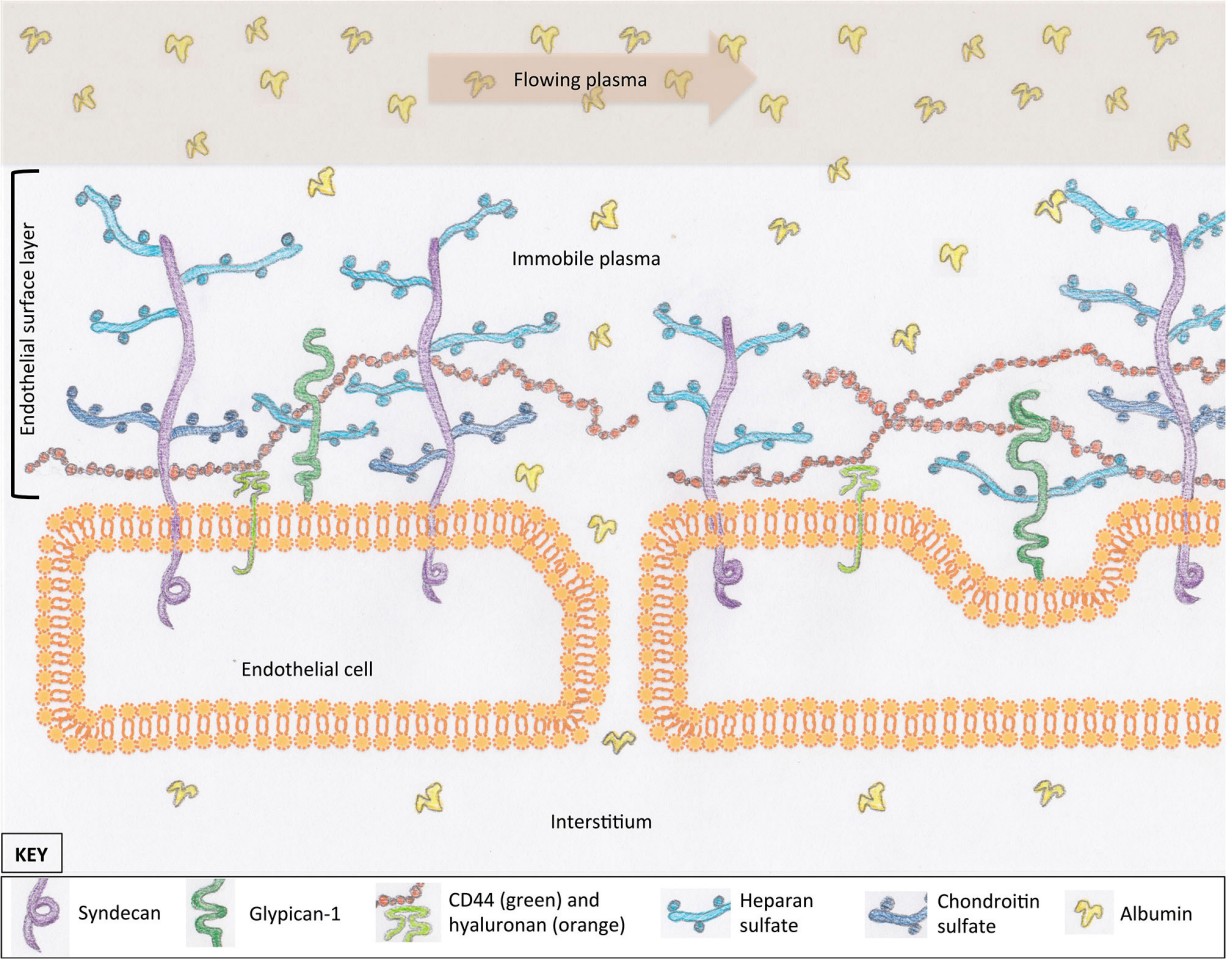
The scaffold of the endothelial glycocalyx, within the endothelial surface layer, is provided by proteoglycans, syndecan (four subtypes), and glypican-1. Glycosaminoglycans are attached to proteoglycans (e.g., heparan sulfate) or the endothelial surface (hyaluronan). Molecules suspended in the plasma of the endothelial surface layer include proteins such as albumin. These proteins create a protein-poor sub-glycocalyx area that is important for transvascular colloid osmotic pressure balance. For simplicity, structures within the interendothelial cleft are not represented.

Most cells in the body are covered in a protective layer of carbohydrate scaffold, which houses many different molecules that serve a variety of functions. This surface layer is called a glycocalyx. The general structure of the glycocalyx has similarities between cell types, with only small variations in individual proteins or carbohydrate molecules. The EG coats the luminal surface of the endothelium and is a vital structure for cell signaling and transvascular permeability. It is composed of proteoglycans, glycosaminoglycans (GAGs), and glycoproteins (Figure 1). Together with mobile or soluble components, such as albumin, this compromises the ESL. Proteoglycans are large molecules that have a cytoplasmic, transcellular, and extracellular domain (syndecan) or are attached by a glycosylphosphatidylinositol anchor (glypican-1) (Figure 1) (10–12). Their extracellular component, or ectodomain, is covered by GAG side-chains and performs important roles that assist with cell to cell, or cell to matrix, interactions (13). These structures provide a structural scaffold for the ESL, in which many other molecules are housed. The sulfated GAGs attached to proteoglycans include heparan, chondroitin, and dermatan sulfate. Heparan sulfate is the most abundant GAG on syndecans and glypican-1, which is why proteoglycans are often referred to as heparan sulfate proteoglycans. An additional GAG, hyaluronan, is not typically associated with a proteoglycan; instead, it is attached to the endothelium via receptors such as CD44 or other GAG molecules (Figure 1) (14, 15). Hyaluronan is a long GAG of varying lengths that weaves its way through the tall “forest” of proteoglycans, with their sulfated GAG “branches”. These GAG chains contribute to the barrier function of the ESL of excluding large molecules (16). Glycoproteins reside on the luminal surface of the endothelium, hidden within the ESL, and include adhesion molecules such as integrins and selectins (17). Glycoproteins play an important role in leucocyte trafficking during states of inflammation (18); many of their functions are only initiated once the ESL has been shed or thinned. Finally, mobile components residing within the forest of the ESL include proteins, such as albumin, and anticoagulants, such as antithrombin and tissue factor pathway inhibitor (15, 19). The presence of plasma proteins are likely important for maintaining the normal structure and permeability of the ESL, as well as providing an anticoagulated surface at the blood interface.

The EG serves a range of functions, many of which are still being characterized. These functions include maintenance of a surface barrier that buffers circulating leucocytes, inhibits coagulation, regulates fluid flux, and communicates changes in vascular wall shear forces. The sulfated GAGs on the surface of the endothelium generate and maintain a net negative charge, which repels similarly charged cells from the endothelial surface (20). Although albumin has an overall negative charge, it is likely that the positively charged groups within the molecular structure are what allow for incorporation of albumin into the ESL (21). Maintenance of this negative charge is important for endothelial integrity; loss of negative charge on the luminal surface of the endothelium leads to extravasation of albumin (22–24). In addition to the importance of electrostatic charge, the ESL provides a barrier to fluid filtration, thus creating a protein-poor sub-glycocalyx layer and maintaining a colloid osmotic pressure (COP) gradient favoring fluid retention within the vasculature (20).

The traditional Starling hypothesis describes a relationship between the intravascular and the interstitial colloid osmotic pressure and implies the importance of this relationship in determining net transvascular fluid filtration. Many studies in the last four decades, however, have built a body of evidence stating that it is mainly the low COP in the sub-glycocalyx area that creates the pressure gradient opposing capillary hydrostatic pressure (25, 26). The theory is that the sub-glycocalyx fluid space, within the inter-endothelial cleft, has a very low concentration of macromolecules. This is due to the high impermeability of the ESL to macromolecules and the rapid flow of water and solutes through the sub-glycocalyx space (27). In the steady state with an intact ESL, net filtration of fluid occurs across the blood vessel wall, with only a small limitation from intravascular COP. Increasing intravascular COP does not, and cannot, serve to reverse net fluid transudation nor does increasing interstitial COP lead to an increase in fluid transudation (28, 29). A large drop in hydrostatic pressure, such as that may occur during circulatory shock, may reverse transvascular fluid flow to a resorptive state; however, this effect is transient (29). These hypotheses explain why fluid would not be “drawn” out of the interstitium by increasing intravascular COP, such as the use of synthetic colloid fluids. Rather, they can reduce extravasation compared to crystalloid fluid. The COP of the intravascular space, and even of the sub-glycocalyx space, is microanatomically and physiologically distant from the interstitial COP. However, the fluid dynamics across the endothelium in disease states with a denuded ESL, removing the influence of the sub-glycocalyx layer, is yet to be fully characterized.

The EG also plays an important role to changing pressures and flow within the intravascular space. Proteoglycans, especially those with heparan sulfate chains, play an important role in responding to changes in vascular wall shear stress or changes in intravascular pressure (10, 30, 31). Detection of these mechanical forces leads to morphological changes in endothelial cells and release of nitric oxide (32–35). Shear stress can also lead to a change in location of proteoglycans or upregulation of their cell surface expression (36–38). It is possible that change in location or upregulation of these molecules during microcirculatory disturbances may also affect shedding of the extracellular components. This becomes relevant when discussing biomarkers for EG shedding below.

### Shedding

Shedding, or modification, of the ESL is a vital step after tissue injury in order to facilitate leucocyte and platelet adherence (39). Shedding reproduced in cell culture or *ex vivo* models leads to increased expression of adhesion molecules, increased leucocyte adhesion, and increased cytokine production (32, 40–43). This process is likely more complex *in vivo*, whereby certain disease states may have varying effects, from selective removal of glycocalyx components to complete denudation. Although ESL shedding is a necessary step in localized inflammation, it is becoming clearer that systemic-wide shedding is associated with severity of illness and poor outcome. There has also been a growing concern in recent years that interventions that promote ESL shedding may worsen clinical outcomes for critically ill patients. Given that ESL shedding is associated with exacerbating inflammation and increasing vascular permeability in laboratory models, there is theoretical plausibility that limiting ESL shedding may improve clinical outcome.

Shedding alters capillary perfusion, causing a decrease in functional capillary density (44), in addition to increased endothelial permeability (45). A decrease in functional capillary density means that some vessels within a given area do not have red blood cells traversing their course, consistent with microcirculatory dysfunction. This can create regions of tissue hypoxia. A clear association between ESL shedding and impairment of microcirculatory blood flow is yet to be demonstrated in either an *in vivo* animal model or clinical study (46). However, given that ESL shedding via artificial means can reduce capillary blood flow (44), it is mechanistically plausible that shedding of the ESL in critical illness plays a role in altered microcirculation. It has been well-demonstrated that heterogeneity of capillary blood flow can persist in critical illness despite normalization of macrohemodynamic variables, such as blood pressure and cardiac output (47–51). Further, persistence of altered microcirculatory flow in critically ill people is associated with severity of illness and poor outcome (47, 51–53). Consequently, it appears important to identify causes of microcirculatory dysfunction that occurs independent of circulatory shock. More evidence is required to determine a causal link between ESL shedding and microcirculatory dysfunction, and whether protection of the ESL can prevent the latter.

There may be downstream consequences to the release of EG components into circulation. Shed components can stimulate inflammation by acting as danger-associated molecular patterns or “alarmins.” Soluble heparan sulfate molecules play a key role in modulating inflammation, including leukocyte activation, increasing production of cytokines, and endothelial activation (54, 55). Low molecular weight hyaluronan can also stimulate production of inflammatory mediators (56–59). In contrast, shed components such as syndecan-1 and−4 ectodomains can have indirect anti-inflammatory effects by facilitating neutrophil cytotoxicity (60, 61). The complexity of EG components acting as effector molecules in the systemic circulation may be analogous to the systemic inflammatory response, where some cytokine release is beneficial to the host response, whereas a “cytokine storm” creates pathological consequences.

It is unclear how long it takes for ESL recovery to occur. Most evidence is based on data from *in vitro* cell culture or *ex vivo* vascular models, which are unable to fully replicate *in vivo* conditions. That being said, individual components can be regenerated within 24 h (37, 62), but restoration of the structure itself can take up to 7 days (32, 63). During critical illness, inflammation likely continues the shedding process, delaying the repair. Further *in vivo* research is required using real-time videomicroscopy or similar means in order to characterize the temporal changes during ESL recovery.

### Assessment of Shedding

Shedding of the ESL can be detected by a number of means. Laboratory studies utilize several methods, including measurement of circulating components of the EG (proteoglycan ectodomains and GAGs), detection of EG components on the surface of the endothelium, direct visualization of the ESL via tissue fixation and microscopy, and indirect visualization via real-time videomicroscopy. Clinical studies usually rely on measurement of circulating components of the EG, or EG biomarkers, in serum or plasma samples to assess systemic shedding. The most frequently reported EG biomarkers are syndecan-1, heparan sulfate, and hyaluronan. There are several limitations to relying on this kind of assessment of the ESL. Firstly, studies often only measure a single component of the EG at a single point in time. That particular component may have other sources of shedding. For example, syndecan-1 is not only present on the surface of the endothelium but also on epithelial cells and leucocytes (64–68). Also, highly relevant to intravenous fluid therapy, hyaluronan is abundant throughout the interstitium and can be “flushed” through the lymphatics back into systemic circulation (69–71). It is unclear in critical illness to what degree other sources of glycocalyx shedding contribute to serum or plasma concentrations. Further, tissue injury and inflammation during critical illness may upregulate cell surface expression of these biomarkers, especially the proteoglycans (38, 72–83). Therefore, an increase in circulating concentration may reflect an increase in cell turnover rather than primarily cell surface shedding. Several recent studies have demonstrated temporal differences in the shedding of several EG components in people presenting to an emergency department with sepsis, whereby hyaluronan increased early in treatment whereas sydnecan-1 increased later (84–86). This raises questions as to why some biomarkers increase earlier than others. It is possible that the temporal differences relate to either alternative sources of the biomarker or differences in upregulation.

Ideally, it would be best to visualize shedding of the EG *in vivo* rather than interpreting circulating biomarker concentrations. These techniques are mostly reserved for use in laboratory models rather than clinical use. Specialized tissue fixation techniques can be used to directly visualize the ESL via electron microscopy, often employed in non-survival rodent models (28, 87–91). Given the fragility of the ESL, it is best that the tissue is preserved via perfusion with fixative prior to death, either perfusion of an isolated organ or the whole body. Tissue immersion techniques that avoid whole-body perfusion have also been recently described (92). Anecdotally, it is challenging to achieve quality images of intact ESL in tissue sections. Alternatively, components of the EG may be fluorescently labeled and visualized by confocal microscopy of cell culture or tissue models (93). Other laboratory techniques include dye exclusion with intravital microscopy to estimate ESL thickness (94–96). Finally, ESL thickness may be estimated in real-time by sidestream dark field microscopy in both large animal models and clinical research (46, 97–99). This technology approximates ESL thickness by measuring the perfused boundary region, which is the region within the blood vessel that is peripheral to the flow of red blood cells, or the immobile plasma layer. Although this technique shows promise for future use in veterinary clinical research (100), image acquisition can be technically difficult and image quality is currently limited for reliably estimating ESL thickness.

## Mechanisms of Shedding Relevant to Fluid Therapy

### Inflammation and Shock

In the patient requiring bolus fluid therapy, it is likely that alteration of the ESL has already occurred due to the effects of inflammatory mediators. For example, tumor necrosis factor-α, a potent pro-inflammatory cytokine, causes reduction in thickness of the ESL, shedding of syndecan-1,-4, and GAGs from the endothelium, as well as upregulation of glycocalyx components on the endothelial surface (76, 81, 90, 101). Matrix metalloproteinases, one of the main perpetrators for glycocalyx shedding, are released by activated leucoytes (17). Tissue injury, such as ischemia and reperfusion, and production of reactive oxygen species also cause EG shedding (43, 102–104). Activation of coagulation may also affect the EG, as both plasmin and thrombin can cleave syndecan ectodomains (74, 105). Relevant to sepsis, bacterial components such as lipopolysaccharide and chemotactic peptides also cause shedding of the EG (43, 77, 81, 106–108). Therefore, it is likely that partial or complete denudation of the ESL has already occurred before fluid therapy is administered.

Shedding of the EG in states of critical illness, such as shock, has been demonstrated in animal models before any fluid therapy has commenced. In mice, hemorrhagic shock was associated with a thinner pulmonary ESL, as measured by electron microscopy, and downregulation of syndecan-1 on the endothelial surface, compared to a sham model (109, 110). In a canine hemorrhagic shock model comparing different fluid interventions (111), plasma hyaluronan concentration was significantly increased across treatment groups after atraumatic blood removal, but before fluid, compared to baseline (unpublished analysis). An increase in plasma hyaluronan concentration has also been demonstrated in a rodent sepsis model, 4 h after *Escherichia coli* lipopolysaccharide infusion, where blood pressure was maintained by norepinephrine administration (112). There are limited data on EG shedding in the clinical setting before fluid therapy; however, serum syndecan-1 concentrations above healthy control levels has been demonstrated pre-hospital in human trauma patients, though the temporal relationship to fluid administration by first responders is unknown (113).

### Hemodilution

Bearing in mind that alterations to the ESL may already exist in critically ill patients, there is growing evidence that administration of “clear” fluids may propagate ESL shedding. Several clinical studies in critically ill people have identified an association between volume of fluid administered and EG biomarker concentrations, including increased hyaluronan (84), syndecan (114), and heparan sulfate concentrations (115). Putting aside the confounders of inflammation and severity of illness, bolus fluid therapy may have a direct effect on the ESL via hemodilution and production of natriuretic peptides. *Ex vivo* vascular models provide important data in regards to this issue, as they exclude the effects of the varying pharmacodynamic properties of fluid types administered *in vivo*. These vascular models have shown that dilution of plasma with crystalloid reduces ESL thickness. Notably, a mathematical model derived from meta-analysis of several studies demonstrated that dilution of blood with fluid reduced vascular resistance, independent of hematocrit and COP (116). Also, the decrease in resistance caused by saline was reduced in magnitude when the vasculature was pre-treated with heparinase. Heparinase sheds heparan sulfate side-chains from proteoglycans, thus thinning the glycocalyx (117). From this, it may be inferred that the decrease in resistance caused by saline infusion is due to not only changes in hemorrheology but also loss of the ESL. Further, several studies have shown that perfusion of blood vessels with crystalloid solution increases vascular permeability. One such study showed an inverse linear relationship between albumin concentration in the perfusate and hydraulic conductivity, or permeability, of vasculature (118). Return to baseline permeability was not achieved by simply increasing the albumin concentration back to baseline levels; it required a much higher albumin concentration. This implies modification of the ESL and its affinity for albumin after crystalloid infusion. Further experiments showed that perfusion of blood vessels with plasma was more effective at restoring normal vascular permeability after crystalloid infusion (119), compared with an albumin solution, indicating that substances within plasma are important for maintaining normal transvascular fluid flux. A more recent study showed that infusion of guinea pig coronary vasculature with 0.9% saline significantly increased fluid extravasation, compared to low molecular weight (LMW) hydroxyethyl starch (HES) (Voluven®) and albumin solution (45). Pretreatment with heparinase combined with saline infusion did not yield higher fluid extravasation; stripping the EG before infusion was just as detrimental as saline infusion alone. Interestingly, no differences between fluid treatments in the appearance of the ESL were appreciated using electron microscopy. Finally, a murine hemorrhagic shock model compared 15 mL/kg of either fresh whole blood (FWB), packed red blood cells in lactated Ringer's solution (PRBC in LRS), or washed PRBC in LRS, or 75 mL/kg of plain LRS (120). Rats that received either washed PRBC or LRS had decreased ESL thickness compared to baseline, as measured by intravital microscopy, whereas those that received either FWB or PRBC did not show a significant difference to baseline. Results were similar for change in plasma heparan sulfate concentration. Therefore, it appeared that the presence of protein within the FWB and unwashed PRBC products was effective at mitigating ESL shedding.

### Natriuretic Peptides

Another mechanism of EG shedding during bolus fluid therapy is the action of natriuretic peptides on the endothelium. Atrial and brain natriuretic peptides (ANP and BNP, respectively) are released from the cardiac atria and ventricles during stretch of the myocardium (121). These peptides counteract the effects of hypervolemia by decreasing systemic vascular resistance and causing a natriuresis, among other mechanisms. Natriuretic peptides also increase vascular permeability (122, 123). A more recently discovered role of these peptides is shedding of the EG. All three major natriuretic peptides (ANP, BNP, and C-type NP) can shed the EG, as measured by increased syndecan-1 and heparan sulfate concentrations in the effluent of guinea pig coronary arteries (88, 124). These studies also demonstrated increased vascular permeability and visualized denudation of the ESL via electron microscopy.

Fast intravenous administration of isotonic crystalloid fluid may stimulate natriuretic peptide release during the bolus phase. The dose of crystalloid administered for treatment of shock far exceeds the volume remaining in circulation after redistribution. This rapid rise and fall of blood volume during a crystalloid bolus was demonstrated by Silverstein and others (7), whereby the blood volume rapidly rose by 76% during a large bolus of 0.9% saline before dissipating. Therefore, it is possible that atria may become “over-stretched” during crystalloid bolus fluid therapy and release natriuretic peptides, which may then contribute to systemic EG shedding. Two studies in human surgical patients have shown a rise in ANP in parallel with EG biomarker concentrations after fluid loading (125, 126). These studies showed a significant increase in serum syndecan-1 and hyaluronan concentrations but not heparan sulfate. One additional study showed an increase in ANP and all three EG biomarker concentrations in cardiac surgical patients; in both those undergoing cardiopulmonary bypass and those undergoing “off-pump” procedures (127). In contrast, one study in people undergoing hysterectomy (*n* = 26) showed no significant rise in BNP or biomarker concentrations (syndecan-1 and heparan sulfate) after 25 mL/kg of LRS given over 30 min during surgery (128). An additional study showed no significant rise in BNP or any EG biomarker concentrations (syndecan-1, heparan sulfate, hyaluronan) in human surgical patients or healthy volunteers during a modest fluid load of 3 mL/kg of 20% albumin, though sample size was small (*n* = 15 per group) (129). A recent canine hemorrhagic shock study did not observe a significant increase in plasma ANP concentration after 80 mL/kg of balanced isotonic crystalloid given over 20 min, however small sample size (*n* = 6) and baseline variability may have hampered the ability to detect a difference (111).

### Other Considerations Related to EG Biomarker Type

One of the challenges of interpreting studies that measure EG biomarker concentrations as the primary assessment of EG shedding is the variability in choice of biomarker. Across multiple studies investigating the effect of fluid loading on the EG, it appears that hyaluronan is consistently increased immediately after a large volume of crystalloid, or synthetic colloid fluid in the setting of hypervolemia (111, 125, 126). Syndecan-1 concentration significantly increases after fluid in humans and rats (110, 125, 126, 130–132), but not in all studies (128, 133). Heparan sulfate has not been shown to significantly increase in humans (125, 126, 128) but has been shown to increase in rats (120, 130, 133). Unpicking these consistencies is difficult due to differences across species, study designs, fluid doses, timing of intervention and blood sampling, and choice of comparator (other fluid vs. no fluid vs. sham). Studies may also vary in whether or not they adjust the biomarker concentration for the effects of hemodilution, such as indexing to albumin, hemoglobin, or other tracer concentration in the blood. Although this may help to account for the different pharmacodynamics of each fluid type, and their variable dilution of blood tracer components, indexation of biomarker concentrations can create a margin of error. On closer inspection of individual studies, inconsistency has been shown within the studies themselves, showing a significant change in one biomarker but not the other (125, 126, 133). As mentioned above, the source of circulating EG biomarkers may not be restricted to the endothelium and other sources, such as the interstitium or surface of other cells, may be contributing. Therefore, a healthy sceptism should be applied to any conclusion drawn from biomarker concentrations alone.

## Comparative Effects of Different Fluid Types

Much of the evidence concerning EG or ESL shedding after fluid therapy concerns the use of large volumes of crystalloid fluid. Administering other fluid types that have less redistribution to the interstitium may theoretically be associated with less EG shedding. This section assesses the evidence for the comparative effect of crystalloid, synthetic colloid, and hypertonic fluids on the EG or ESL.

### Isotonic Crystalloid Fluid

Multiple rodent studies have shown that administration of large volumes of crystalloid fluid for hemorrhagic shock is associated with more EG shedding, compared to fluids containing protein (109, 110, 120, 130–133). However, it is difficult to compare interventions in regards to effects on the EG in many of these studies, as cardiovascular parameters are either not closely monitored or are different between treatment groups. Shock itself, without fluid resuscitation, can cause EG shedding (109, 110, 131, 134–136), which becomes a confounder when comparing resuscitation strategies that provide inequitable blood volume expansion. However, several key studies allow some assessment of this issue. A murine hemorrhagic shock model showed that administration of protein-poor fluids, either washed PRBCs or LRS, was associated with a thinner ESL, whereas protein-containing fluids (FWB and PRBCs) were not (120). Cardiovascular parameters after resuscitation appeared similar across these groups, based on the limited data available. Another murine hemorrhagic shock model compared seven different fluid strategies: 15 mL/kg of FWB, PRBC, fresh frozen plasma (FFP) or 5% albumin, 8 mL/kg of 3% saline, 45 mL/kg of 0.9% saline, or 75 mL/kg of LRS (130). Shock index, usually defined by heart rate divided by systolic blood pressure, was compared before hemorrhage and immediately after fluid resuscitation within each fluid group. Rats that received either 5% albumin, LRS, 3% saline, or 0.9% saline had a significantly increased shock index after fluid resuscitation, whereas rats that received FWB, PRBC, and FFP showed no change in shock index. When comparing only the fluid types that did not resolve shock, based on shock index, rats that received 5% albumin had higher ESL thickness and lower EG biomarker concentrations, compared to the crystalloid groups. Finally, a murine hemorrhagic shock model used a blood pressure-targeted resuscitation method for comparing crystalloid fluid with plasma (109). Administration of LRS was associated with a thinner ESL, visualized on electron microscopy, and decreased endothelial expression of syndecan-1, compared to administration of plasma.

There is a scarcity of “large animal” models, or those including pigs, sheep, and dogs, that have assessed ESL shedding after fluid resuscitation. This may be partially due to a limitation on validated assays available for measuring glycocalyx biomarkers across these species. Current commercially available validated options for assessing EG shedding are restricted to circulating hyaluronan concentration. A canine hemorrhagic shock model did not detect a significant difference in plasma hyaluronan concentrations when comparing dogs that received 20 mL/kg of FWB (*n* = 6) with those that received 80 mL/kg of Plasmalyte-148® (*n* = 6) (111). Other differences in parameters were seen between the crystalloid and colloid fluid groups in this study, which is discussed further below. In an ovine endotoxemia model, a pressure-targeted resuscitation method using vasopressor therapy was used to compare either norepinephrine alone or 0.9% saline (40 mL/kg) in combination with norepinephrine (137). Although there was no comparison to resuscitation with a protein-containing fluid, the comparison of crystalloid fluid to *no* fluid in the setting of sepsis provided interesting results. There was no significant difference in serum hyaluronan concentrations between groups over time, though sheep that received 0.9% saline showed a greater rate of increase in this biomarker. This greater increase was not observed directly after the fluid bolus, like in the aforementioned canine study, but beyond 6 h, which may have been related to worsening septic shock in this group. Atrial natriuretic peptide was significantly higher at the end of the fluid bolus in the sheep that received fluid (see previous discussion on natriuretic peptides). Detection of between-group differences was also likely affected by small sample size in this study (*n* = 8 per group), similar to the study in dogs.

### Synthetic Colloid Fluids

Given that crystalloid fluids generally require a larger volume to expand circulating blood volume to the same extent as colloid fluids (6–9), crystalloids cause greater hemodilution during the fluid bolus and, potentially, greater natriuretic peptide release. This begs the question if administration of colloid fluids causes less EG shedding than crystalloid fluid. Two main types of synthetic colloid are currently in use: HES and gelatin products. The aforementioned canine hemorrhagic shock study included both of these fluid types at 20 mL/kg, as comparator fluids to 80 mL/kg of Plasmalyte-148® (111). Dogs administered HES had significantly lower plasma hyaluronan concentration immediately after the fluid bolus, compared to both FWB and crystalloid groups. In contrast, the group that received 4% succinylated gelatin had significantly increased plasma hyaluronan concentration 40 and 100 min after the end of the fluid bolus, compared to FWB. Given that inflammation may cause EG shedding, these differential effects observed between these two colloids may be due to release of inflammatory mediators; HES has been associated with mitigation of inflammation (138–141) whereas gelatin has been associated with pro-inflammatory effects (142). However, there were no differences in plasma concentrations of inflammatory mediators between the two colloid groups in this study (111). Therefore, the effect of gelatin on hyaluronan shedding may be a direct effect of the fluid, rather than pro-inflammatory effects, such as washout of the interstitium. Another hemorrhagic shock study in rats replaced the shed volume with crystalloid, at either shed volume, two times shed volume, or three times shed volume, or shed volume with HES (*n* = 6 per group) (141). This study did not identify any significant differences between groups in response of blood pressure to fluid resuscitation, organ syndecan-1 expression, or circulating syndecan-1 concentration. A hemorrhagic shock, stroke volume-targeted resuscitation model in pigs compared balanced crystalloid to HES and found no differences in post-fluid serum syndecan-1 or glypican concentrations (6). Blood was sampled 120 min after commencement of fluids, therefore any peaks in EG biomarker shedding may have been missed. Given the limited evidence for different effects of colloid fluids on the EG or ESL, compared to crystalloids, a conclusion cannot be currently drawn.

### Hypertonic Crystalloid Fluids

Hypertonic solutions exert immunomodulatory effects both in *in vitro* and animal model studies, including decreased leucocyte response to pro-inflammatory stimuli or circulatory shock states, when compared to other fluid types (143–156). Administration of hypertonic saline to human trauma patients also reduced inflammatory biomarker concentration, compared to isotonic saline (157, 158). Several studies have also shown a reduction in vascular leakage of macromolecules (147, 151, 155). Given these effects, it is possible that hypertonic solutions may have a beneficial effect in regards to reducing ESL shedding. In the rodent hemorrhagic shock model previously discussed comparing many different type of fluids, rats that received 8 mL/kg of 3% saline had a lower increase from baseline in plasma heparan sulfate concentration, compared to both isotonic crystalloid groups (45 mL/kg of 0.9% saline or 75 mL/kg of LRS), and a lower loss of ESL thickness (130). This difference was despite a persistence of an elevated shock index and hyperlactatemia after resuscitation with 3% saline. Therefore, although hypertonic saline may have had less impact on the EG, it may have also provided inadequate fluid resuscitation. A preliminary randomized clinical trial in people with suspected sepsis could not demonstrate a difference between groups in hyaluronan concentrations after treatment allocation of either 5 mL/kg of 3% saline or 10 mL/kg of 0.9% saline (86). Serum syndecan-1 concentration decreased after 0.9% saline, compared to baseline, whereas serum syndecan-1 concentration did not change after 3% saline. Though this significant difference between groups may reflect hemodilution, as twice the volume of 0.9% saline was administered, this was not reflected in differences in hemoglobin concentration. It is unclear what the clinical significance is of this subtle difference in change in serum syndecan-1 concentration, whether it reflects more syndecan-1 shedding in the hypertonic saline group or not. Limitations of this study included a low severity of illness and small sample size, and further studies are needed to clarify the effects of hypertonic solutions on the EG.

## Glycoprotective Therapies

The clinical relevance of ESL shedding due to fast or large-volume fluid administration is yet to be determined. This type of fluid administration can cause shedding of the ESL, which may propagate microcirculatory dysfunction, inflammation, and procoagulation. Therefore, there has been much interest developing in “glycoprotective” fluid strategies or adjunctive therapies during the resuscitation phase.

### Restricting or Avoiding Fluid

Blood volume expansion is the cornerstone of managing circulatory shock, with some types of shock an exception such as cardiogenic shock. Conventionally, this is achieved by administering large (more than 20 mL/kg) volumes of crystalloid fluid intravenously as rapidly as possible, or as a bolus (159, 160). This serves to increase cardiac preload and improve cardiac output. The need for this intervention is intuitive for shock caused by hypovolemia; however, some types of shock have a component of vasodilation and microcirculatory dysfunction. This includes states of shock due to a systemic inflammatory response, such as sepsis and blunt trauma. For these types of shock, improvement of macrohemodynamic variables, such as cardiac output and blood pressure, may not improve microcirculatory blood flow in a linear way. This is particularly pertinent to septic shock, where the role of fluid bolus therapy has been called into question, with a movement toward early vasopressor therapy instead (161, 162). Further to the lack of improvement in microcirculatory flow, it has been suggested that fluid bolus therapy in sepsis can propagate vasodilation, due to endothelial shear stress, release of nitric oxide, and direct vasodilatory effects of natriuretic peptides. This may contribute to some patients becoming refractory to vasopressor therapy. In an ovine endotoxemia model, administration of 40 mL/kg of 0.9% saline was associated with a significantly higher dose of noradrenaline subsequently administered in order to maintain blood pressure, compared to sheep that received only noradrenaline (137). Given the concern that bolus fluids may cause harm in sepsis, a preliminary randomized clinical trial was completed demonstrating feasibility of restricting crystalloid fluid early in the treatment of sepsis in people (163). Following on from this, a large multi-center human randomized clinical trial is underway to compare a restrictive fluid strategy with a more liberal one in people with early sepsis (164).

It is much less known if restricting crystalloid fluid in other types of shock may be beneficial. In the setting of human trauma, discussion concerning restrictive crystalloid fluid strategies focuses on delayed resuscitation or permissive hypotension in order to avoid clot disruption and dilutional coagulopathy before definitive hemostasis (4, 165). Although animal models have shown that fluid resuscitation is likely beneficial for severe uncontrolled hemorrhage, it increases the risk of mortality in lower grades of severity of bleeding, compared to not administering any fluid (166). Hypotensive resuscitation, in parallel with lower volumes of administered fluids, is also associated with a reduced risk of mortality in human trauma patients (167, 168), though the evidence is mixed (169). This benefit is likely related to improved coagulation and clot stability; it is unknown if there are also benefits associated with reducing endothelial dysfunction. Currently, the recommendation in human trauma medicine is for limitation of “clear” fluid and early, yet judicious, administration of blood products. This includes packed red blood cells, plasma, and platelets in parallel during resuscitation. The early administration of plasma may not only serve to limit dilutional coagulopathy but also assist with repairing the ESL. It is difficult to translate this practice to veterinary medicine, given the cost of transfusion, limited availability, and variability in blood banking practices. Given there are many factors entering into the cost to benefit assessment of administering plasma or albumin products to individuals, theoretical benefits of plasma for the ESL are of lesser importance, until more is known. Also, caution should be applied when restricting crystalloid fluid to any patient with shock, as the benefits of a restrictive strategy may be only relevant to certain patient populations, such as sepsis. This concept was highlighted by a recent randomized clinical trial in human surgical patients whereby participants randomized to receive a restrictive fluid strategy had a significantly higher rate of acute kidney injury and surgical site infection, compared to those randomized to a liberal fluid strategy (170).

### Slowing Fluid Administration

Fast fluid administration serves to rapidly improve clinical signs and macrohemodynamic variables, and has been the bread-and-butter of emergency and critical care medicine for decades. However, normalization of macrohemodynamic variables in people does not always translate to improved microcirculatory flow (47–49, 51). This is termed a lack of hemodynamic coherence (171). Intravenous fluid delivered rapidly during the stabilization phase may be contributing to persistence of disturbed microcirculatory flow. When viewing the microcirculation using sidestream dark field microscopy during anesthesia in pigs, fast fluid administration (20 mL/kg/hr) for 3 h was associated with development of heterogeneity in capillary blood flow, compared to “standard” anesthesia fluid rates (5 mL/kg/hr) (172). Although a direct relationship between estimated ESL thickness and microcirculatory flow has not been established in the clinical setting (46), it is possible that amplification of ESL shedding caused by fast fluid administration may be contributing to microcirculatory dysfunction. It is possible that slower fluid administration may achieve the same resuscitation end-points while avoiding some of these deleterious effects on the endothelium. This concept has been explored in a handful of studies. In a rodent model where 40% of the blood volume was removed by atraumatic hemorrhage, rats were assigned to receive no fluid, rapid fluid (0.9% saline at three times shed volume over 30 min), or slow fluid (0.9% saline at three times shed volume over 12 h) (173). Rats in the fast fluid group showed higher inflammatory cytokine concentrations early in the study and some markers of worse outcome over the latter part of the study, including lower mean arterial blood pressure, higher blood glucose and lactate concentrations, and an increase in markers of lung injury, compared to rats in the slow fluid group. As the rats were euthanased after 24 h, it is unknown how their recovery progressed. In a human open randomized clinical trial, including 50 surgical and 16 septic patients, no significant difference was found in estimated ESL thickness between patients that received fast crystalloid administration (5–10 min) and slow crystalloid administration (20–30 min) (48). However, an overall decrease in estimated ESL thickness after fluid administration was observed. Given that there is some evidence that rapid fluid administration (i.e., within 10 min) may not provide any clinical benefit over slower fluid administration (i.e., within 20 to 60 min) (8, 174), larger clinical trials are justified to explore effects of fluid rate on the microcirculation and clinical outcomes.

### Choice of Clear Fluid Type

Intense ongoing debate surrounds the choice of fluid for critically ill patients, including crystalloid, synthetic colloid, and protein-containing solutions. These conversations surround relative fluid effectiveness and adverse effects, many of which are still yet to be fully elucidated for clinical relevance in certain species or disease subsets. Adding in the potential benefit or detriment of certain fluid types to preservation of the ESL and optimizing microcirculation is still premature. Bearing that in mind, the current evidence supports mitigating hemodilution and avoiding hypervolemia in order to optimize ESL recovery. There is theoretical benefit for the ESL to the use of protein-containing solutions, such as plasma and albumin, rather than “clear” fluids, but this must be carefully considered against any detrimental effects of blood products, including financial cost. At this stage, no clear recommendation can be made for veterinary medicine, and more clinical research is required.

### Adjunctive Therapies

Many drugs have been explored as to their protective or resurrecting actions for the ESL and have been reviewed in detail elsewhere (175, 176). These therapies may become viable in the future for use during the optimization and stabilization phase of fluid resuscitation (177). Therapies that are in current use in veterinary medicine that have been shown to have beneficial effects on the EG or ESL include albumin solution, plasma, hydrocortisone, heparin, and N-acetylcysteine.

## Conclusions

The coating of endothelium by the EG and its associated molecules serves many functions in the body, both intact and when denuded. Infusion of large volumes of fluid causes disruption of this layer, which may propagate interstitial edema and inflammation. There is some evidence that slow fluid administration or restricting the volume of crystalloid fluid for shock resuscitation may benefit the patient. Exactly how to do this and for which patient subset this strategy benefits in veterinary medicine remains to be identified. Clinical veterinary research assessing the effect of glycoprotective therapy or strategies is currently limited by a lack of validated EG biomarker assays and affordable, reliable technology for estimating ESL thickness *in vivo*. Further work is required on developing validated, reliable EG biomarker assays and determining the relationship between these biomarkers and shedding of the ESL *in vivo*, especially for dogs and cats. Despite these current limitations, future research directions should also focus on strategies limiting crystalloid fluid volumes, especially in the setting of sepsis and early vasopressor drug therapy.

## Author Contributions

LS: preparation of manuscript and final approval. DH: revision of manuscript and final approval. Both authors contributed to the article and approved the submitted version.

## Conflict of Interest

The authors declare that the research was conducted in the absence of any commercial or financial relationships that could be construed as a potential conflict of interest.
